# The diversity of *Klebsiella pneumoniae* surface polysaccharides

**DOI:** 10.1099/mgen.0.000073

**Published:** 2016-08-25

**Authors:** Rainer Follador, Eva Heinz, Kelly L. Wyres, Matthew J. Ellington, Michael Kowarik, Kathryn E. Holt, Nicholas R. Thomson

**Affiliations:** ^1^​ LimmaTech Biologics AG, Schlieren, Switzerland; ^2^​ The Wellcome Trust Sanger Institute, Hinxton, Cambridge, UK; ^3^​ Centre for Systems Genomics, University of Melbourne, Parkville, Victoria, Australia; ^4^​ Department of Biochemistry and Molecular Biology, Bio21 Molecular Science and Biotechnology Institute, University of Melbourne, Parkville, Victoria, Australia; ^5^​ National Infection Service Public Health England, London, UK; ^6^​ London School of Hygiene and Tropical Medicine, London, UK

**Keywords:** Klebsiella pneumoniae, seroepidemiology, surface polysaccharide, K antigen and O antigen, vaccine target

## Abstract

*Klebsiella pneumoniae* is considered an urgent health concern due to the emergence of multi-drug-resistant strains for which vaccination offers a potential remedy. Vaccines based on surface polysaccharides are highly promising but need to address the high diversity of surface-exposed polysaccharides, synthesized as O-antigens (lipopolysaccharide, LPS) and K-antigens (capsule polysaccharide, CPS), present in *K. pneumoniae*. We present a comprehensive and clinically relevant study of the diversity of O- and K-antigen biosynthesis gene clusters across a global collection of over 500 *K. pneumoniae* whole-genome sequences and the seroepidemiology of human isolates from different infection types. Our study defines the genetic diversity of O- and K-antigen biosynthesis cluster sequences across this collection, identifying sequences for known serotypes as well as identifying novel LPS and CPS gene clusters found in circulating contemporary isolates. Serotypes O1, O2 and O3 were most prevalent in our sample set, accounting for approximately 80 % of all infections. In contrast, K serotypes showed an order of magnitude higher diversity and differ among infection types. In addition we investigated a potential association of O or K serotypes with phylogenetic lineage, infection type and the presence of known virulence genes. K1 and K2 serotypes, which are associated with hypervirulent *K. pneumoniae*, were associated with a higher abundance of virulence genes and more diverse O serotypes compared to other common K serotypes.

## Data summary

Supplementary dataset S1 lists the ENA accession numbers for the 573 publicly available *K. pneumoniae* whole-genome Illumina read sets analysed in this study (http://www.ebi.ac.uk/ena).

Representative *rfb* and *cps* locus sequences have been deposited in GenBank, see Table S2 for the list of GenBank accession numbers (http://www.ncbi.nlm.nih.gov/genbank/).

## Impact statement

Vaccines offer a potential remedy against the increasing threat of pan-drug-resistant *Klebsiella pneumoniae* strains. However the high diversity of surface antigens poses a challenge for vaccine design. This work is the first, to our knowledge, to catalogue naturally occurring polysaccharide antigen biosynthesis gene clusters in a globally representative collection of *K. pneumoniae* isolates, enabling us to identify novel serotypes and perform an epidemiological analysis. We show that only three O-antigen serotypes account for the majority of infections, offering a promising target for vaccine design.

## Introduction

*Klebsiella pneumoniae* is a leading cause of hospital- and community-acquired infections (including urinary tract infections, pneumonia, bacteraemia and soft tissue infections), primarily afflicting the young and immunocompromised, despite being part of the normal human intestinal microbiota and able to colonize the skin and nasopharynx of healthy individuals (Podschun & Ullmann 1998; Broberg *et al.* 2014). *K. pneumoniae* are naturally resistant to antibiotics such as amino-penicillins and carboxy-penicillins. Increasingly, treatment options are diminishing, leaving third-generation cephalosporins and carbapenems as the remaining alternative. However, the emergence of isolates carrying genes encoding extended spectrum beta lactamases (ESBL) and carbapenemases has raised alarm because it removes these last line treatment options and effectively negates the use of a whole class of antibiotics, with few alternatives. Therefore multiple agencies including the WHO, US Centers for Disease Control and Prevention and the UK Department of Health singled out *K. pneumoniae* as a global health concern (Boucher *et al.* 2009; Ahmad *et al.* 2012).

Historically *Klebsiella* isolates have been classified into serotypes and tracked using typing antisera. Serotyping is based on the recognition of distinct variations of surface-exposed polysaccharides, namely O-antigens and K-antigens, by specific antibodies, resulting in different O and K serotypes. O-antigens are the outermost part of the lipopolysaccharide (LPS), whereas K-antigens belong to the bacterial capsule polysaccharide (CPS). The number of serotypes has been estimated to be eight for O-antigens and 77 for K-antigens (Orskov, Fife-Asbury 1977; Trautmann *et al.* 1997; Edwards, Fife 1952; Edmunds 1954).

Multivalent protein-conjugate polysaccharide vaccines have been demonstrated to be highly successful and effective against bacterial pathogens, such as *Streptococcus pneumoniae* (Center, 2007). In *K. pneumoniae*, polyvalent vaccines based on the K-antigen have been developed and reached Phase I trials in humans (Edelman *et al.* 1994; Campbell *et al.* 1996). However, the high diversity of K-antigens and the confusing seroepidemiology render a vaccine with a broad coverage complex to develop and thus very costly. Compared with other *Enterobacteriaceae*, such as *Escherichia coli* [161 defined O serotypes (Iguchi *et al.* 2015a)] and *Shigella flexneri* [at least 47 O serotypes (Talukder *et al.* 2003)], *Klebsiella* has a surprisingly low number of reported O serotypes which promises a more viable alternative for vaccine development compared with K-antigen-based vaccines (Ahmad *et al.* 2012). Whole-genome sequence data allows us to verify and assess the diversity of these clusters and also estimate their frequency in contemporary isolates to assess the contribution of isolates bearing these markers.

The O-antigen biosynthesis enzymes are encoded on the *rfb* locus. To date, seven O-antigen clusters have been defined for *K. pneumoniae*, associated to serotypes O1, O2, O3, O4, O5, O8 and O12 (Table S1) and the *rfb* O-antigen biosynthetic pathway is well described (Raetz & Whitfield, 2002; Kalynych *et al.* 2014; Whitfield & Trent 2014). In essence it is an ABC-transporter-dependent pathway functionally composed of three types of enzymes: those responsible for (i) biosynthesis of nucleotide-activated sugars, (ii) polysaccharide repeat-unit synthesis and (iii) assembly of the repeat units and transport across the membrane (flippases).

**Fig. 1.**
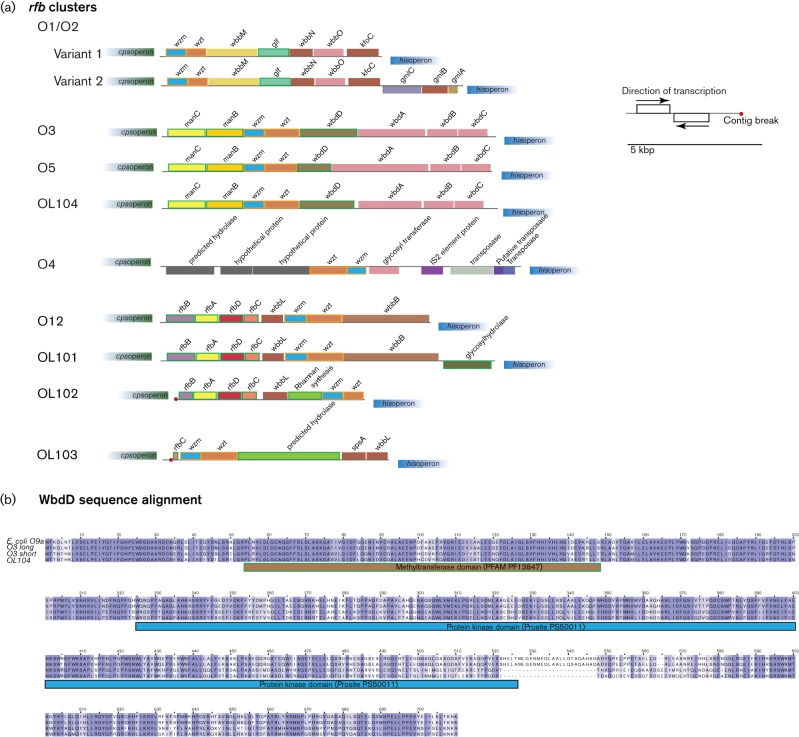
(a) *rfb* gene clusters (see Fig. S1 , available in the online Supplementary Material for full list), genes are colored according to function (see Fig. S1) and above or below the line according to the coding strand and the direction of transcription (black arrows). (b) WbdD sequence alignment. Comparison of *E. coli* 09a (Uniprot accession number Q47592) and one representative each for the two types of *K. pneumoniae* O3 WbdD (O3 long (isolate 5151_3#1) and O3 short (9878_1#12)) and OL104 wbdD (5193_7#2). The alignment was colored according to the BLOSUM62 score. Domain detection was performed using InterProScan5 (Jones *et al.* 2014) based on the *E. coli* WbdD sequence.

Both the O1 and O2 antigen polysaccharide chains are based on a repeat-unit designated as d-galactan I. The difference being that the O1 antigen is capped by a distal d-galactan II unit, whereas O2 is not (Vinogradov *et al.* 2002; Kol *et al.* 1992). d-galactan II is the only known O-antigen polysaccharide in *Klebsiella* for which biosynthesis is enabled by genes (*wbbY* and *wbbZ*) unlinked to the *rfb* cluster (Hsieh *et al.* 2014). The O1/O2 biosynthetic cluster occurs in two variants (Fig. 1a). Variant 1 consists of the transporter genes (*wzm* and *wzt*), three glycosyltransferases responsible for repeat-unit synthesis and an UDP-galactopyranose mutase, responsible for the synthesis of the polysaccharide subunit d-galactan I (Clarke & Whitfield 1992). Variant 2 is extended and carries three additional putative glycosyltransferases encoded by *gmlABC*. These additional genes are thought to explain the existence of a recently defined O2 subtype whereby the *gml* gene products modify d-galactan I, to give the chemically and antigenically distinct d-galactan III explaining the different subtype (Kelly *et al.* 1995; Szijártó *et al.* 2015). The serology of O1 isolates is proposed to be unchanged by GmlABC, because in this instance d-galactan II (encoded by WbbY and WbbZ) is the dominant antigenic epitope (Szijártó *et al.* 2015).

O-antigen polysaccharide structure and *rfb* sequence of *K. pneumoniae* serotypes O3 and O5 are identical to those of *E. coli* serotypes O9a and O8, respectively (Vinogradov *et al.* 2002; Iguchi *et al.* 2015b). Biosynthesis enzymes of O3 and O5 differ in the sequence of their mannosyltransferase (WbdA) and methyltransferase (WbdD). WbdD, in complex with WbdA, regulates the mannose chain length by capping the growing chain with a phosphate and methyl group in O3 and a methyl group only in O5 (Hagelueken *et al.* 2012, 2015).

For completeness the O8 polymer is identical to the O1 antigen, with the exception of the partial O-acetylation of the d-galactan I repeat unit in O8 (Kelly *et al.* 1993) and serotype O9 may be considered as a subtype of serogroup O2 (Hansen *et al.* 1999; Trautmann *et al.* 1997; Kelly & Whitfield 1996). Serotype O9 Wzm and Wzt sequences share >95 % identity to the O8 Wzm and Wzt orthologues (Fang *et al.* 2015)

Whilst most members of the *Enterobacteriaceae* have one conserved LPS core, two types of outer cores have been reported for *K. pneumoniae*; they differ in a set of three genes within the *waa* operon (Regué *et al.* 2005), which is independently located in the genome.

The seroepidemiology of *K. pneumoniae* has previously shown that for human-host-associated isolates the most prevalent O serotypes are O1, O2 and O3, with O1 being dominant in human disease (Trautmann *et al.* 1997; Hansen *et al.* 1999; Yu *et al.* 2007; Fang *et al.* 2015). The observed prevalence of O4, O5 and O12 differs among the studies, whereas the significance of serotypes O8 and O11 remain equivocal (Trautmann *et al.* 1997). For O11 this is due to the lack of available *rfb* sequences and publicly available O11 isolates, so they could not be considered in this study. In addition, little is known about the association between O serotype and disease presentation, the only exception being the observation that serotype O3 isolates are more commonly isolated from blood samples compared with urine samples (Trautmann *et al.* 1997; Hansen *et al.* 1999).

The K-antigen biosynthesis enzymes are encoded on the *cps* (capsule polysaccharide synthesis) locus. To date the *cps* gene clusters of the 77 serologically defined K-types and nine additional distinct *cps* operons have been identified and published (Pan *et al.* 2015; Chung The *et al.* 2015; Wyres *et al.* 2015). The biosynthesis pathway is a Wzy-dependent polymerization pathway, identical to *E. coli* Group 1 capsule synthesis (Whitfield 2006; Larue *et al.* 2009; Shu *et al.* 2009; Bushell *et al.* 2013).

There is some confusion in the literature regarding the prevalence of K serotypes and their association with disease outcome (Cryz *et al.* 1986; Fung *et al.* 2000, 2002; Yu *et al.* 2007). This may be explained by the higher diversity of the K serotypes and possible cross-reactivity, making them much harder to define. In some studies K1 and K2 showed the highest prevalence and were associated with poorer disease outcome (Fung *et al.* 2000, 2002; Yu *et al.* 2007). In addition, these K serotypes have been associated with the emergence of hypervirulent *K. pneumoniae* causing community-acquired invasive infections such as pyogenic liver abscess, which has become of particular concern in parts of Asia (Shon *et al.* 2013). Hypervirulent K1 strains generally belong to the ST23 lineage, while K2 is found in more diverse backgrounds (Struve *et al.* 2015; Bialek-Davenet *et al.* 2014). However, in many studies outside of Asia, including an extensive seroepidemiological study of European and North American cases, K1 isolates are rare (Cryz *et al.* 1986).

Until now our knowledge of the nature and diversity of O- and K- operons and, by proxy, the antigens they encode is based on sequences of a limited number of reference isolates. Recently, the whole-genome sequences of more than 500 isolates collected from environmental samples, plants, mammals and non-human primates, as well as those from asymptomatic human carriage, from cases of invasive disease and from both the clinical and community setting were sequenced and published (Holt *et al.* 2015; Ellington, 2016; Chung The *et al.* 2015; Wand *et al.* 2015) (Table 1). These studies highlighted that the ability to cause invasive infections is not determined by lineage but is associated with the presence of virulence factors such as siderophore systems and the *rmpA* mucosity factor, although these virulence determinants are overrepresented in lineages associated with the hypervirulence phenotype (Holt *et al.* 2015; Bialek-Davenet *et al.* 2014; Struve *et al.* 2015).

**Table 1.** Genome data included in this study and references

Dataset	Note	Reference
Global	289 isolates; Human and environmental isolates, from six countries (Australia, Indonesia, Laos, Singapore, Vietnam, USA), sampled to maximize diversity and exclude members of a clonal outbreak, metadata includes invasiveness, acquisition type and sample site. Invasiveness status of human isolates consists of three types: carriage (isolates not considered to be the cause of an infection), non-invasive (pneumonia, urinary tract infection, wound infection; with no recorded bacteraemia) and invasive (isolated from normally sterile sites: blood, CSF, intra-ocular, pleural, pericardial, joint fluids, deep-seated tissue abscesses). Acquisition type is either community acquisition (isolated within 48 hours of admission to hospital) or hospital acquisition (isolated after 48 hours after admission)	(Holt *et al.* 2015)
UK Hospital	162 isolates; Collection from Cambridge University Hospitals NHS Foundation Trust in the UK over a period of seven years, invasive isolates isolated from normally sterile sites. Biased selection for antimicrobial resistance to three or more of six antimicrobial classes (penicillins, amoxacillin-clavulanate, aminoglycosides, fluoroquinolones, trimethoprim and third-generation cephalosporins), metadata includes sample site	(Ellington 2016)
Nepal Hospital	88 isolates; Nepalese hospital outbreak from May to December 2012, consisting mainly of two clonal lineages; randomly selected blood cultures.	(Chung The *et al.* 2015)
Preantibiotic	34 isolates; Collection of isolates isolated before the widespread use of antibiotics (pre 1949). No additional metadata available.	(Wand *et al.* 2015)

Using this comprehensive whole genome dataset we set out to describe the genetic diversity of O-antigen and K-antigen and LPS core biosynthesis gene clusters and classify them by molecular serotyping. Using these data we then set out to determine if there was an association between O-, K- or LPS core types and disease outcome, phylogenetic lineage or the presence of other known virulence-related genes.

## Methods

### Bacterial isolates.

Publicly available genome data derived from four different *K. pneumoniae* collections were analyzed in this study, totaling 573 sequenced isolates (Table 1). The global dataset consists of a globally representative collection from six different countries including isolates from different hosts and different infection types (Holt *et al.* 2015). The UK hospital dataset consists of a collection from the Cambridge University Hospitals NHS Foundation Trust and contains invasive isolates biased towards those with resistance to third-generation cephalosporins collected over a period of seven years (Ellington 2016). The Nepal hospital dataset contains human isolates from a single Nepalese hospital outbreak in 2012 (Chung The *et al.* 2015). The preantibiotic dataset contains strains isolated before the widespread use of antibiotics (pre 1949) (Wand *et al.* 2015).

Genome data, generated in the above studies by paired end Illumina sequencing, were sourced from the European Nucleotide Archive (accession numbers are listed in Supplementary dataset S1, available in the online supplementary material). Reads were *de novo* assembled using Velvet (Zerbino & Birney 2008) and Velvet Optimiser, and the resulting assemblies annotated using Prokka (Seemann 2014), as described previously (Holt *et al.* 2015).

### *In silico* serotyping.

O serotyping was performed on the basis of the polysaccharide ABC transporters (flippases; Wzm and Wzt). The specificity of transporters to polysaccharide types has been noted previously (Cuthbertson *et al.* 2007). The protein sequences of Wzm were located and extracted from the assembled and annotated contigs using tblastn (Camacho *et al.* 2009) and grouped based on sequence clustering using CD-HIT (Fu *et al.* 2012) with an identity threshold of 95 %. This threshold was empirically derived and shown to be able to differentiate between the previously described *rfb* clusters (Table S1. The resulting groups were assigned the O serotype of known Wzm sequences (Table S1), which were included in the clustering step. In cases where Wzm was missing, Wzt homology was used for assignment. Any *rfb* loci which differed from previously described loci in their sequence and gene content were assigned to putative novel O serotypes. Novel *rfb* loci sequences were given the designation OL[n], etc, (OL=LPS locus, to differentiate those defined on the basis of *rfb* locus sequences from those defined serologically; *n* is a numeric identifier beginning from *n*=100) (Fig. S1, Table S2). The differentiation of O1 from O2 serotypes was based on the presence of WbbY and WbbZ in O1 (NCBI accession number KJ451390, strain NTUH-K2044 (Hsieh *et al.* 2014)). LPS core types were assigned as described above, using WaaL clustering with an identity threshold of 80 %. The *waa* gene cluster was extracted by locating WaaC and WabG using tblastn. Several operons contained a contig break or stretches of low sequencing coverage; when no *waaL* could be identified or did not fit into any of the two main groups, these were marked as unassigned.

Due to the much higher diversity of the K antigen, grouping was performed based on the full-length *cps* sequence, which was extracted by locating *wzi* and *wzc* using tblastn (Camacho *et al.* 2009) and extending the presumed locus until a gene on the opposite strand appeared. The extracted *cps* locus DNA sequences were grouped using UCLUST (Edgar 2010) based on a 95 % identity. The groups were assigned to a serotype if they matched to any of the 86 previously described *cps* locus sequences (Pan *et al.* 2015; Chung The *et al.* 2015; Wyres *et al.* 2015).

Additionally, a database of *wzi* (Brisse *et al.* 2013) and *wzc* (Pan *et al.* 2013) sequences with known serotypes was used to verify the classifications. Any *cps* clusters which differed from previously described loci in their sequence and gene content were assigned to putative novel K serotypes. Putative full-length novel clusters were confirmed by generation of alternative assemblies using SPAdes 3.6.1 (Bankevich *et al.* 2012) and inspection of the resulting assembly graph using Bandage (Wick *et al.* 2015). Sequences were confirmed as full length if they spanned from the 5′ *galF* to the 3′ *ugd* gene. Full-length clusters were given the designation KL[n], etc: KL=capsule locus, to differentiate those defined on the basis of *cps* locus sequences from those defined serologically; *n* is a numeric identifier beginning from *n*=101 (although the first two loci (KL101 and KL102) have already been named KN1 and KN2 in the literature, and thus we will continue to use these designations here). Representative nucleotide sequences of full length *cps* and *rfb* clusters were annotated using Prokka (Seemann 2014) followed by manual curation, and deposited in GenBank (Fig. S1, Table S2). Novel *wzi* alleles were added to the *K. pneumoniae* whole-genome sequence typing database BIGSdb at the Institut Pasteur (http://bigsdb.web.pasteur.fr/) (Bialek-Davenet *et al.* 2014).

### Seroepidemiology of human *K. pneumoniae* infections.

To analyze the prevalence of different O and K serotypes in human disease and their putative association with disease outcome, the 216 human-associated isolates of the global collection were used to investigate their seroepidemiology.

Three different infection properties were examined for association between O and K serotypes and LPS core variant: (i) infection site: we focused on isolates sampled from human blood (representing bacteraemia), sputum (pneumonia) and urine (urinal tract infections, UTI), isolates from other sites were excluded from this category, (ii) acquisition type: hospital-acquired isolates had been sampled in patients from 48 h after admission to hospital, community-acquired isolates had been sampled within 48 h of admission to hospital, isolates where no acquisition type was recorded were excluded from this category, (iii) infection status: carriage isolates are intestinal samples without any infection, non-invasive isolates are infections without bacteraemia (such as wound infection, pneumonia, UTI) and invasive isolates are from infections of normally sterile sites (such as blood).

### Gene presence.

A database of putative virulence genes was compiled (Table S3) and the presence of these genes was characterized as described previously (Chung The *et al.* 2015).

### Multi locus sequence typing (MLST).

MLST sequence types for all isolates were determined directly from sequence reads using SRST2 (Inouye *et al.* 2014) to type against the seven-locus MLST scheme (Diancourt *et al.* 2005). Sequence types (ST) are listed in Supplementary dataset S1.

### Comparative gene analysis and phylogenetic trees.

Comparative gene analysis and core genome definition was performed using Roary (Page *et al.* 2015), using a blastp percentage identity of 95 % and a core definition of 99 %.

**Fig. 2.**
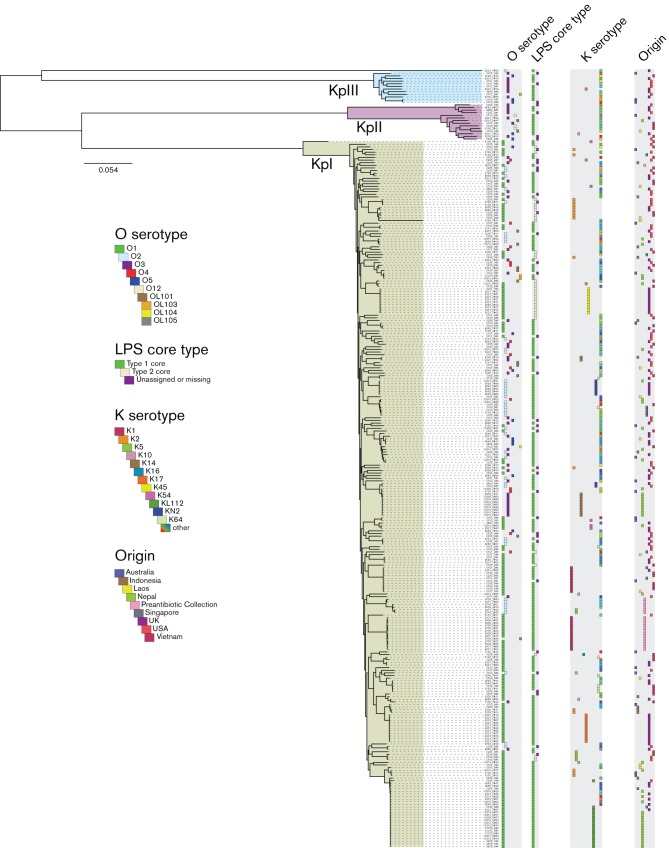
Phylogeny of a randomly selected subset of isolates. Lineages are labeled according to the scheme of (Holt *et al.* 2015), subdividing the *K. pneumoniae* species into subspecies KpI (*K. pneumoniae*), KpII (*K. quasipneumoniae*) and KpIII (*K. variicola*). O and K serotypes and LPS core types, as well as the geographical origin, are denoted by colored squares as indicated.

Phylogenies were inferred from either a SNP alignment generated by mapping reads to the *K. pneumoniae* MGH78578 reference sequence (Fig. S2) or from a concatenated alignment of core genes extracted from Roary output (Page *et al.* 2015) (Fig. 2). Single-gene alignments (Fig. 1b and Fig. S3) were performed using Clustal Omega (Sievers *et al.* 2011). Phylogenies were inferred from these alignments by running RAxML using a gamma distribution to model site-specific rate variation and 100 bootstrap replicates (Stamatakis 2006).

## Results

### Genetic structures of the *rfb* gene cluster

The *rfb* gene cluster was located from the genome sequence data and classified on the basis of Wzm and Wzt protein sequence. The *rfb* cluster was universally located between the *cps* and *his* gene clusters in all of our samples (Fig. S1). It was evident from these data that there were *rfb* operons that did not match any of the known *rfb* gene clusters (i.e. they did not belong to O1, O2, O3, O4, O5, O8 or O12), either as a result of different gene content or differing nucleotide sequence. These clusters were defined as novel geno-/serotypes. Out of the 573 isolates analyzed in this study, 533 isolates could be assigned to six known O serotypes, 36 isolates to five novel serotypes, and four isolates remain unassigned due to contig breaks or missing sequencing coverage (Supplementary dataset S1).

The clusters themselves range in size from seven to ten genes (8–10 kb). Both O1/O2 variants, O3, O4, O5 and O12, and four novel *rfb* clusters (OL101, OL102, OL103 and OL104) were observed in our dataset (Fig. 1a). The novel cluster, OL104, is identical in gene repertoire to O3 and O5, both synthesizing a mannose polymer repeated subunit. OL104 possesses the same WbdA as O3, that contains two mannosyltransferase domains and is distinct from the longer O5 WbdA, that contains three mannosyltransferase domains, which have been shown to have functional consequences in the mannosyl linkages they are able to synthesize (Greenfield *et al.* 2012). Within the isolates designated as serotype O3, two types of WbdD, the chain-length regulator, were observed. One (present in 34 of a total of 86 O3 isolates screened; 40 %) is identical to the *E. coli* O9a WbdD (Uniprot accession number Q47592), whereas the other type (present in the remaining 52 isolates; 60 %) has a deletion of 25 aa at the C-terminal region of its protein kinase domain. The WbdD of the isolates designated as serotype OL104 shows the same deletion (Fig. 1b). We speculate that OL104 and the two O3 WbdD variants might actually represent serologically distinct subtypes of the O3 serogroup.

The *K. pneumoniae* O12 antigen is composed of an N-acetylglucosamine and rhamnose polymer (Vinogradov *et al.* 2002). Based on their gene content and order, O12, OL101 and OL102 are also closely related *rfb* clusters differing by the presence and absence of single genes: Compared with the O12 cluster, OL101 includes an additional glycosylhydrolase located on the opposite strand, while OL102 lacks the terminal glycosyltransferase gene but possesses an additional rhamnan synthesis gene (Fig. 1a).

It is of note that the direction of transcription in all of the *rfb* operons run antiparallel to those genes belonging to the adjoining *his* operon, with exception noted above, the O4 cluster and the extended variant of the O1/O2 O-antigen cluster (O1/O2 Variant 2) (Fig. 1a). The O4 *rfb* cluster is the only *rfb* cluster in *K. pneumoniae* containing a transposase. The O4 antigen is based on a galactose and ribofuranose polymer (Vinogradov *et al.* 2002).

When assessing the genetic diversity of the *waa* LPS core biosynthesis operon we detected both of the known core types, but found no conclusive evidence for novel core types.

### Genetic structures of the *cps* gene cluster

The *cps* gene cluster showed a strikingly higher level of diversity both in sequence and gene content, compared to the *rfb* clusters (Fig. S1). Clusters ranged in size from 20.5 to 21.6 kb.

Of the 573 isolates analyzed, 387 (68 %) were assigned to 52 known K serotypes by comparison to previously described *cps* clusters representing the serologically typed reference strains (Pan *et al.* 2015) (Supplementary dataset S1). Three isolates (0.5 %) where only a partial sequence was available were assigned to a serotype with lower confidence due to only a partial match to the reference sequence. A total of 165 isolates (29 %) for which the *cps* sequence was >5 % different to those of any of the known *cps* clusters were putatively assigned to 68 novel serotypes. Eighteen isolates (3 %) remained unassigned because no *wzi* and *wzc* sequences could be identified in the genomes. Among the putative novel serotypes, 21 distinct full-length *cps* sequences were identified (Fig. S1). Five of these sequences matched or were transposase variants of those previously described in (Wyres *et al.* 2015) and (Chung The *et al.* 2015), including one which was a transposase-negative variant of the *cps* cluster from *K. pneumoniae* HS11286 (genome accession NC_016845.1).

Consistent with results from previous studies, the first gene in all *cps* clusters was *galF* (Ebrecht *et al.* 2015) followed by a putative glucose phosphatase (*cpsACP*), the JUMPstart sequence (‘just upstream of many polysaccharide starts’) followed by the translocation and surface assembly genes (*wzi, wza, wzb* and *wzc*) (Rahn *et al.*, 1999). Although the genes responsible for transport into the periplasm and polymerization (*wzx* and *wzy*), UndPP-linkage (*wbaP* or *wcaJ*) and a 6-phosphogluconate dehydrogenase [*gnd*, a housekeeping gene not required for CPS synthesis (Chen *et al.* 2010)] were universally present, they were not found at fixed locations in these clusters. The *cps* clusters are usually terminated by UDP-glucose 6-dehydrogenase gene (*ugd*). The gene encoding glucose dehydratase (*rffG*) separates the *cps* and *rfb* clusters.

### The distribution of the *rfb* and *cps* gene clusters across *K. pneumoniae* phylogeny

To understand the distribution of the *rfb, cps* and *waa* gene clusters across the *K. pneumoniae* species we reconstructed a whole-genome-based phylogeny for all isolates included in this study and show their distribution (Fig. 2). This highlights that, with some exceptions, *rfb* clusters are not restricted to any particular clade. In our dataset the O1 and O2 *rfb* loci are only found in the KpI sublineage (*K. pneumoniae sensu stricto (*Holt *et al.* 2015)), for the other *rfb* clusters there is extensive evidence of horizontal gene transfer of the different *rfb* clusters between closely and more distantly related lineages. This is further confirmed by the non-concordance of the phylogeny of the *rfb* gene clusters and the whole-genome phylogeny [O1 and O2: Fig. S2(a); O3, O5 and Novel 4: Fig. S2(b)].

The two *waa* LPS core types are equally not restricted to particular clades in the phylogeny, although we note a biased distribution with respect to the O serotypes. Out of the 573 isolates in the full dataset, 70 isolates (12 %) encoded core type 2; these core 2 strains are almost exclusively (65 isolates, 93 %) associated with serotype O1. The remaining isolates are core 1 (446, 78 %) or unassigned (57, 10 %). One previous study (Regué *et al.* 2005) investigated the distribution of LPS core types in 100 *K. pneumoniae* strains based on PCR and dot blots and found a ratio of LPS core 1 to LPS core 2 isolates similar to our data (79 : 19), and also noted the association of LPS core 2 with serotype O1 (10 out of 34 O1 strains); however we did not observe an association with K serotype K2 as has been previously reported. Our analysis shows an elevated number of strains carrying both the LPS core type 2 and K2 or K45, however this is likely to be strongly influenced by two clonal expansions within the sample collection we analysed (Fig. 2). Furthermore, we could only observe LPS core type 2 in subspecies KpI (Fig. 2).

Due to the high diversity, K serotype switching is more difficult to analyze. As an example, K1 isolates belong mainly to two lineages, one of which (ST23) is associated with the hypervirulent phenotype, whereas the K2 serotype is much more distributed across the phylogenetic tree, including lineages associated with the hypervirulent phenotype (e.g. ST25) and other non-hypervirulent lineages (e.g. ST14, Fig. 2). This is consistent with previous reports which showed that many *K. pneumoniae* sequence types include multiple CPS types (Wyres *et al.* 2015; Holt *et al.* 2015; Brisse *et al.* 2009).

### O and K Serotype epidemiology

Looking across the full dataset of the 573 isolates the majority were genotyped as O1 (296 isolates, 52 %), followed by O2 (91, 16 %), O3 (86, 15 %), O5 (33, 6 %), OL101 (26, 5 %), O4 (18, 3 %) and O12 (9, 2 %). Each of the OL102 to OL104 serotypes occur in less than 1 % of isolates. The O1/O2 extended *rfb* cluster variant 2 was observed in 112 (38 %) of O1 genotyped isolates and 52 (57 %) O2 isolates (Supplementary dataset S1).

The seven most common O serotypes described above were associated with 54, 39, 40, 22, 14, 7 and 9 distinct K serotypes respectively, suggesting there is a relationship between the number of representatives of each O serotype in our dataset and the number of distinct K serotypes with which each is associated. This relationship is best explored using only the global isolate set, which is not biased towards any particular *K. pneumoniae* lineage or isolate source. Within this dataset O1 is the most common O serotype (*n*=133) and was associated with 43 distinct K serotypes. O3 is the next most common O serotype (*n*=45), followed by O2 (*n*=39) and O5 (*n*=24), these were associated with 29, 30 and 20 distinct K serotypes, respectively. Among the same isolate set the most common K serotype was K2 (*n*=20), associated with two distinct O serotypes (O1 and O2). K1 (*n*=15) and K64 (*n*=14) are the next most common serotypes and are associated with two and four O serotypes respectively. All other K serotypes are represented by eight or fewer isolates in our collection and were each associated with just one or two distinct O serotypes. These data indicate that re-assortment of K and O types occurs frequently in *K. pneumoniae*.

**Fig. 3.**
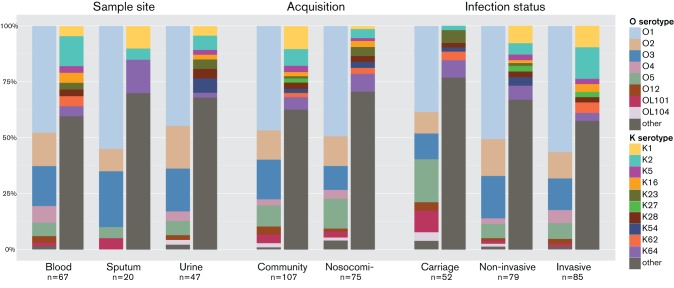
Distribution of serotypes in human *K. pneumoniae* isolates in the global collection. The eight most common O-types (left bars) and ten most common K-types (right bars) are shown. Other serotypes are shown in dark grey.

To investigate the seroepidemiology in human disease, we analyzed the association of O and K serotypes with the three different recorded infection properties (infection site, acquisition type and infection status), using the human-associated isolates of the global collection (Table 2, Fig. 3). Fisher’s exact test was performed to examine whether there was a significant association between serotypes or LPS core types and infection sites, acquisition type or infection status. Significant correlations were found between K2 and invasive infections (odds ratio 4.1, *p*=0.008), and between OL101 O type and asymptomatic carriage (OR 8.5, *p*=0.009) (Table 2). Of special interest is also K1, which was virtually restricted to community-acquired isolates (OR 8.4, *p*=0.016) and completely missing in carriage-associated isolates (*p*=0.024). No correlation was found for any association of either O or K serotype to infection sites and no correlation of LPS core type with any of the traits as above was observed.

**Table 2.** Distribution of serotypes and LPS core types in human *K. pneumoniae* isolates in the global collection (*n*=216) The eight most common O-types and ten most common K-types are shown. In the sample site category, samples from other than blood, urine or sputum were excluded. In the acquisition mode category, samples where no acquisition mode was reported were excluded.

	Sample site						Acquisition				Infectiousness					
O serotype	Blood *n*=67		Urine *n*=47		Sputum *n*=20		Community *n*=107		Nosocomial *n*=75		Carriage *n*=52		Infection *n*=79		Invasive *n*=85	
O1	32	47.8 %	21	44.7 %	11	55.0 %	50	46.7 %	37	49.3 %	20	38.5 %	40	50.6 %	48	56.5 %
O2	10	14.9 %	9	19.1 %	2	10.0 %	14	13.1 %	10	13.3 %	5	9.6 %	13	16.5 %	10	11.8 %
O3	12	17.9 %	9	19.1 %	5	25.0 %	19	17.8 %	8	10.7 %	6	11.5 %	15	19.0 %	12	14.1 %
O4	5	7.5 %	2	4.3 %	0	0.0 %	3	2.8 %	3	4.0 %	0	0.0 %	2	2.5 %	5	5.9 %
O5	4	6.0 %	3	6.4 %	1	5.0 %	10	9.3 %	10	13.3 %	10	19.2 %	5	6.3 %	6	7.1 %
O12	2	3.0 %	1	2.1 %	0	0.0 %	4	3.7 %	1	1.3 %	2	3.8 %	1	1.3 %	2	2.4 %
OL101	1	1.5 %	0	0.0 %	1	5.0 %	4	3.7 %	2	2.7 %	5^†^	9.6 %	1	1.3 %	1	1.2 %
OL104	0	0.0 %	1	2.1 %	0	0.0 %	2	1.9 %	1	1.3 %	2	3.8 %	1	1.3 %	0	0.0 %
Others^∗^	1	1.5 %	1	2.1 %	0	0.0 %	1	0.9 %	3	4.0 %	2	3.8 %	1	1.3 %	1	1.2 %
																
K serotype																
K1	3	4.5 %	2	4.3 %	2	10.0 %	11	10.3 %	1	1.3 %	0	0.0 %	6	7.6 %	8	9.4 %
K2	9	13.4 %	3	6.4 %	1	5.0 %	8	7.5 %	3	4.0 %	1	1.9 %	4	5.1 %	12^† ^	14.1 %
K5	2	3.0 %	1	2.1 %	0	0.0 %	3	2.8 %	1	1.3 %	0	0.0 %	2	2.5 %	2	2.4 %
K16	3	4.5 %	1	2.1 %	0	0.0 %	2	1.9 %	2	2.7 %	0	0.0 %	1	1.3 %	3	3.5 %
K23	0	0.0 %	0	0.0 %	0	0.0 %	1	0.9 %	3	4.0 %	3	5.8 %	1	1.3 %	0	0.0 %
K27	2	3.0 %	2	4.3 %	0	0.0 %	2	1.9 %	0	0.0 %	0	0.0 %	2	2.5 %	2	2.4 %
K28	2	3.0 %	2	4.3 %	0	0.0 %	3	2.8 %	2	2.7 %	1	1.9 %	2	2.5 %	2	2.4 %
K54	0	0.0 %	3	6.4 %	0	0.0 %	2	1.9 %	2	2.7 %	1	1.9 %	3	3.8 %	0	0.0 %
K62	3	4.5 %	0	0.0 %	0	0.0 %	2	1.9 %	2	2.7 %	2	3.8 %	0	0.0 %	4	4.7 %
K64	3	4.5 %	1	2.1 %	3	15.0 %	6	5.6 %	6	8.0 %	4	7.7 %	5	6.3 %	3	3.5 %
Others^∗^	40	59.7 %	32	68.1 %	14	70.0 %	67	62.6 %	53	70.7 %	40	76.9 %	53	67.1 %	49	57.6 %
																
LPS core type																
Type 1	43	64.2 %	35	74.5 %	12	60.0 %	68	63.6 %	55	73.3 %	37	71.2 %	55	69.6 %	55	64.7 %
Type 2	7	10.4 %	3	6.4 %	2	10.0 %	9	8.4 %	10	13.3 %	7	13.5 %	7	8.9 %	11	12.9 %
Others^∗^	17	25.4 %	9	19.1 %	6	30.0 %	30	28.0 %	10	13.3 %	8	15.4 %	17	21.5 %	19	22.4 %

∗ Unidentified or not listed † Significant correlation (see text)

To assess any correlation of serotypes to geographical origins, the dataset was grouped based on acquisition type and infection site in order to remove any spurious relationship. The groups were analyzed as described above; no statistically significant correlation between serotypes or LPS core types and geographical origin could be found. No significant correlation of the presence of O1/O2 *rfb* cluster extended variant 2 to infection site, infection type or sample site was found.

### Association between serotype and virulence gene content

**Fig. 4.**
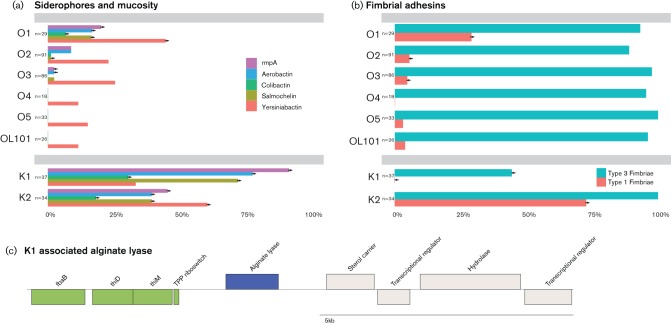
(a,b) Virulence genes in *K. pneumoniae* serotypes. Frequency of isolates containing (a) siderophore and mucosity and (b) fimbrial adhesin genes for selected serotypes. →, Significantly overrepresented in this serotype vs all other serotypes (Fisher’s exact test, *P*-value <0.01). ←, Significantly underrepresented. (c) Genomic context of the K1 associated alginate lyase (blue: alginate lyase, green: thiamine pyrophosphate biosynthesis cluster, grey: other).

Multiple virulence factors have been defined for *Klebsiella* (Lawlor *et al.* 2007; Broberg *et al.* 2014) including three siderophores (yersiniabactin, salmochelin and aerobactin), the cytotoxin colibactin and *rmpA/rmpA2*, a regulator of CPS overexpression (Cheng *et al.* 2010). Fimbrial adhesins are also known to play a major role in biofilm formation and are thus also classified as virulence factors. We determined if the distribution of these known virulence functions was correlated with O- or K-serotype using the existing global data set. The most noteable association was the significant enrichment of an array of siderophore and colibactin clusters and the mucosity regulator *rmpA* with K serotypes K1 and K2 (Fig. 4a). Since K1 and K2 isolates were almost exclusively also O1 serotypes, the same effect was observed in O1. These results are perhaps unsurprising given that these virulence genes are overrepresented in the hypervirulent *K. pneumoniae* lineages, which are also associated with serotypes K1 and K2 (Holt *et al.* 2015; Bialek-Davenet *et al.* 2014).

Two types of fimbriae are known to be present in *K. pneumoniae*: type 1 and type 3 fimbriae. Whilst type 3 fimbriae are known to bind to different human cell types, ranging from tracheal epithelia to renal tubular cells, as well as being important for binding to plants and abiotic surfaces (Khater *et al.* 2015), type 1 fimbriae appear more specific, so far they have been shown to be important for adhesion to uroepithelial cells and also for binding to plants (Klemm, Schembri 2000). Both type 1 and type 3 fimbriae are also associated with biofilm formation in *K. pneumoniae* (type 3 fimbriae more strongly than type 1 (Schroll *et al.* 2010; Stahlhut *et al.* 2012; Klemm, Schembri 2000). Our data showed that serotype O1 isolates had a significantly higher likelihood of possessing type 1 fimbriae compared with any other O serotype (odds ratio 9.7, *p*=1.1E-16×E-16E-1610^–16^, Fisher’s exact test) (Fig. 4b). Differences in type 1 and type 3 fimbriae occurrence are very pronounced between K1 and K2: whereas K1 lacks type 1 fimbriae genes (none present, *p*=0.0019), K2 has a significantly higher occurrence compared with any other K serotype (OR 15.8, *p*=3.82×10^–13^); type 3 fimbriae genes are rarer in K1 (OR 0.03, *p*=6.43×10^–17^) compared with any other K serotype. The majority of K1 isolates possessing neither type 1 nor type 3 fimbriae (20 isolates without type 1 or type 3 fimbriae out of a total of 35 K1 isolates), stem from a single sequence type (ST82) present in the preantibiotic collection.

To further investigate the fact that K1 occurs almost exclusively in community-acquired isolates and is absent in nosocomial infections, a comparative genomic analysis was performed to search for genes that are overrepresented in K1 compared with other K-serotypes. Strikingly, an alginate lyase isozyme was found to be present in all K1 isolates and virtually absent in all other K serotypes (*p*=4.82×10–57, Fisher’s exact test). This K1-associated alginate lyase is located just upstream of the thiamine pyrophosphate (TPP) biosynthesis cluster (Fig. 4c). The only other commonly occurring alginate lyase gene in the dataset was present within the K14 *cps* cluster and has a sequence identity to the K1 associated alginate lyase below 45 %. Alginate lyases enable the use of alginate as carbon and energy source (Wong *et al.* 2000).

## Discussion

The *rfb* and *cps* gene clusters give rise to the dominant serotypical properties of *K. pneumoniae* and are therefore priority vaccine candidates for the treatment of this increasingly multidrug-resistant pathogen. We have catalogued the naturally occurring diversity of these gene clusters within a large collection of isolates taken from different geographic and clinical settings, hosts and disease manifestations. In doing so we identified six known and five novel O-antigen clusters and 45 known and 18 novel complete K-antigen biosynthesis gene clusters. Of particular interest is a previously undescribed *rfb* cluster (OL101), occurring in 5 % of isolates in our dataset, originating in Europe, Asia and North America and found in human, bovine and environmental samples. We also identified a further 46 putative *cps* clusters (for which only partial sequences could be obtained) which appear novel. Although this may be an overestimate as some of these may represent transposase variants or divergent forms of other *cps* types or result from assembly errors; additional work will be required to investigate and validate these further.

This genetic catalogue of *rfb* and *cps* clusters enabled *in silico* serotyping of whole genome sequences. We focused on the seroepidemiology by analyzing the human-associated isolates from the global dataset (Holt *et al.* 2015), where clinical parameters such as infection site, type and acquisition mode have been recorded. We showed that O serotypes O1, O2 and O3 accounted for 80 % or more of all samples included in this study and that the relative prevalence of these O serotypes was approximately the same for all infection sites, infection types and acquisition modes. This contrasted with the K serotypes, for which an order of magnitude higher diversity was found and no single K serotype dominated this collection.

Notable in the K serotype analysis was the distribution of serotype K1 sequences: most K1 isolates in our study belong to one of two lineages, ST23 and ST82 and showed a strong association with community-acquired infections, consistent with previous reports and the known association of ST23 with the hypervirulent phenotype (Tsay *et al.* 2002; Yu *et al.* 2007). K1 isolates were found in six out of seven countries covered by our genome collection. However, despite the fact that K1 is regarded as one of the two most common serotypes it was only found once in 162 isolates collected over a period of seven years in a UK hospital (this study) and is absent in another study based on 703 isolates in 13 hospitals located in Western Europe and Northern America (Cryz *et al.* 1986). This contrasts with reports from Taiwan, China and South Africa where K1 appears to have been dominant for a considerable period of time (Fung *et al.* 2000,^2002^; Yu *et al.* 2007; Peng *et al.* 1991; Luo 1990) and has been associated with comparatively higher prevalence of hypervirulent infections in these countries, mostly linked to the ST23 lineage (Bialek-Davenet *et al.* 2014).

When looking for genes closely associated with K1 we identified the presence of a gene predicted to encode an alginate lyase isozyme. Outside of K1 the only other alginate lyase detected is distantly related and part of the K14 *cps* cluster. Hence, this gene is almost exclusively found in isolates of the K1 serotype (including those of both major K1 lineages). *Pseudomonas aeruginosa* is known to produce alginate during biofilm biogenesis in chronic lung infections of cystic fibrosis patients. Alginate lyase enables cell detachment from the biofilm (Boyd & Chakrabarty 1995; Ramsey & Wozniak 2005). However it is not known whether *K. pneumoniae* produces alginate biofilms. Moreover, since it is unusual to find alginate-lyase-producers, like the K1 *K. pneumoniae*, that do not use alginate as primary carbon source, it has been proposed that alginate lyase production could be related to coinfection of *P. aeruginosa* and *K. pneumoniae* in cystic fibrosis patients (Wong *et al.* 2000). Given the above it is suggested that the association with cystic fibrosis is important, but probably it is opportunistic, and its role in *K. pneumoniae* is much broader.

We found no association of LPS core type with the isolates' infection sites, infection types and acquisition modes. This is in contrast to a study showing that LPS core type 2 contributes to the level of virulence in *K. pneumoniae*, although its mechanism is so far unknown (Regué *et al.* 2005).

Virulence genes (siderophores, colibactin, *rmpA* and fimbriae) are expected to be overrepresented in isolates from infections compared with those from carriage or environmental sources and are particularly overrepresented in lineages associated with hypervirulent invasive disease (Holt *et al.* 2015), thus the significantly high presence in *K. pneumoniae* serotype O1 isolates was anticipated. However, their significantly lower abundance in isolates of serotype O2 or O3, which are both also prevalent in human infection, was more surprising.

The striking difference in the distribution of the two major fimbriae types found in *K. pneumoniae* is also of note. Type 3 fimbriae are essential for biofilm formation (Schroll *et al.* 2010), while type 1 fimbriae are important for adhesion to uroepithelial cells and are thus considered virulence factors for urinary tract infection (Stahlhut *et al.* 2012). The differential distribution of fimbriae in *K. pneumoniae* probably allows isolates to adhere to different receptors and perhaps exploit or specialize in different niches, the presence of type 1 fimbriae in K2 isolates and the notable absence of type 1 fimbriae in K1 isolates is intriguing but will require more targeted sampling to unravel its true biological significance.

It is suspected that certain CPS types are able to influence the accessibility of the LPS O-antigen, possibly masking it; the reports are however inconclusive: Hsieh *et al.* (2012) described the masking of the O1 antigen by K1 capsule but not by K2, whereas Szijártó *et al.* (2015) showed that the O1 antigen is accessible by antibodies irrespective of the capsule type. The potential masking of O-antigen by the capsule warrants further research and is the crucial next step towards a polysaccharide-based vaccine against *K. pneumoniae* infection.

Our data indicate that O and K serotypes are frequently re-assorted within the *K. pneumoniae* population. There are far fewer distinct O serotypes than K serotypes but among the O serotypes, those that are most common are generally associated with a higher number of distinct K serotypes. While there are lineage-specific clustering effects (Fig. 2), these data support the hypothesis that the *rfb* and/or *cps* clusters are shuffled within the *K. pneumoniae* population via horizontal gene transfer. However given the wide diversity of K serotypes, much larger strain collections will need to be examined in order to detect a statistically significant divergence from random re-assortment of CPS and LPS loci.

*K. pneumoniae* is considered to be a significant threat to human health with the rates of infection increasing globally and appearing to be driven by increasing levels of antimicrobial resistance to front line antimicrobials. The short-term solution has been to turn to old drugs such as colistin that are associated with significant nephrotoxicity. However, the development of resistance to colistin is rapid and explained by both intrinsic mechanisms, such as point mutations (Cheng *et al.* 2015), as well as by the acquisition of genes by lateral gene transfer (Liu *et al.* 2015). With this in mind we found that O-serotype prevalence and distribution were stable with regards to different infection types and sites. In addition although O serotype switching has been a common occurrence in the evolution of *K. pneumoniae* K serotypes show a much bigger variance (Wyres *et al.* 2015). Consequently O antigens, defined in detail in this study, offer a promising target for vaccine design that warrant further research.

## Supplementary Data

95 Supplementary File 1

## Supplementary Data

37 Supplementary File 2

## Supplementary Data

17 Supplementary File 3

## Supplementary Data

14 Supplementary File 4

## Supplementary Data

1891 Supplementary File 5

## Supplementary Data

263 Supplementary File 6

## Supplementary Data

437 Supplementary File 7
