# Supplementary material for: The diversity of Klebsiella pneumoniae surface polysaccharides
Source: Microb Genom. 2016 Aug 25;2(8):e000073. doi: 10.1099/mgen.0.000073 (PMC5320592; doi:10.1099/mgen.0.000073)
Supplement: Supplementary file 2 [file mgen-02-73-s002.docx]

**Supplementary Table S1**: Known *rfb* operon sequences of O serotypes

| **Serotype** | **LPS structure** | **NCBI Acc number** | **Strain** | **Reference** |
| --- | --- | --- | --- | --- |
| O1 | (1) | AB117611 | NTUH-K2044 | (2) ^a^ |
|  |  | AB819964 | Friedlander 204 | (3) ^b^ |
|  |  | JQGN01 (Assembly) | 325 | (Unpublished, University of Malaya) ^b^ |
| O2 | (1) | AB795943 | 5053 | (3) ^b^ |
| O3 | (1) | AB010295 | O3:K53 | (4) ^c^ |
|  |  | AB010296 | O3:K49S | (4) ^c^ |
|  |  | AB795941 | 636/52 | (3) ^b^ |
| O4 | (1) | KU310493 | Mich. 61 | (3) ^b^ |
| O5 | (1) | AB819962 | 5710/52 | (3) ^b^ |
| O8 | (7) | AB819963 | 889 | (3) ^b^ |
| O9 | (7) | Not published, only wzm and wzt available | 1205 | (3) ^d^ |
| O12 | (1) | AB795942 | 702 | (3) ^b^ |

^a^ Strain serotyped by (5)

^b^ Strain serotyped by (6)

^c^ Strain serotyped by (4)

^d^ Strain serotyped by (8), suspected to be an O2 subtype (6)

References

1. **Vinogradov E, Frirdich E, MacLean LL, Perry MB, Petersen BO, Duus JØ, Whitfield C.** 2002. Structures of lipopolysaccharides from Klebsiella pneumoniae. Eluicidation of the structure of the linkage region between core and polysaccharide O chain and identification of the residues at the non-reducing termini of the O chains. The Journal of biological chemistry **277**(28)**:**25070–25081. doi:10.1074/jbc.M202683200.

2. **Fang C, Chuang Y, Shun C, Chang S, Wang J.** 2004. A novel virulence gene in Klebsiella pneumoniae strains causing primary liver abscess and septic metastatic complications. The Journal of experimental medicine **199**(5)**:**697–705. doi:10.1084/jem.20030857.

3. **Fang C, Shih Y, Cheong C, Yi W.** 2015. Rapid and Accurate Determination of Lipopolysaccharide O-Antigen Types in Klebsiella pneumoniae with a Novel PCR-Based O-Genotyping Method. Journal of clinical microbiology. doi:10.1128/JCM.02494-15.

4. **Sugiyama T, Kido N, Kato Y, Koide N, Yoshida T, Yokochi T.** 1998. Generation of Escherichia coli O9a serotype, a subtype of E. coli O9, by transfer of the wb* gene cluster of Klebsiella O3 into E. coli via recombination. Journal of bacteriology **180**(10)**:**2775–2778.

5. **Hsieh P, Lin T, Yang F, Wu M, Pan Y, Wu S, Wang J.** 2012. Lipopolysaccharide O1 antigen contributes to the virulence in Klebsiella pneumoniae causing pyogenic liver abscess. PloS one **7**(3)**:**e33155. doi:10.1371/journal.pone.0033155.

6. **Hansen DS, Mestre F, Alberti S, Hernández-Allés S, Alvarez D, Doménech-Sánchez A, Gil J, Merino S, Tomás JM, Benedí VJ.** 1999. Klebsiella pneumoniae lipopolysaccharide O typing: revision of prototype strains and O-group distribution among clinical isolates from different sources and countries. Journal of clinical microbiology **37**(1)**:**56–62.

7. **Kelly RF, Whitfield C.** 1996. Clonally diverse rfb gene clusters are involved in expression of a family of related D-galactan O antigens in Klebsiella species. Journal of bacteriology **178**(17)**:**5205–5214.

8. **Trautmann M, Ruhnke M, Rukavina T, Held TK, Cross AS, Marre R, Whitfield C.** 1997. O-antigen seroepidemiology of Klebsiella clinical isolates and implications for immunoprophylaxis of Klebsiella infections. Clinical and diagnostic laboratory immunology **4**(5)**:**550–555.
