# Supplementary material for: The diversity of Klebsiella pneumoniae surface polysaccharides
Source: Microb Genom. 2016 Aug 25;2(8):e000073. doi: 10.1099/mgen.0.000073 (PMC5320592; doi:10.1099/mgen.0.000073)
Supplement: Supplementary file 3 [file mgen-02-73-s003.docx]

**Supplementary Table S2:**

GenBank accession numbers of submitted representative O- and K-type sequences

| **K- or O-type** | **GenBank acc #** | **Based on isolate number** |
| --- | --- | --- |
| O1/O2 Variant 1 | LT174601 | 10315_6#10 |
| O1/O2 Variant 2 | LT174602 | 10315_6#74 |
| O12 | LT174600 | 9221_7#92 |
| O3 wbd long | LT174603 | 9221_7#18 |
| O3 wbd short | LT174604 | 10315_6#92 |
| O4 | LT174605 | 5150_5#1 |
| O5 | LT174606 | 9663_8#14 |
| OL101 | LT174596 | 10315_6#47 |
| OL102 | LT174597 | 5197_2#10 |
| OL103 | LT174598 | 5151_2#7 |
| OL104 | LT174599 | 5193_5#1 |
| K1 | LT174541 | 9776_2#25 |
| K10 | LT174532 | 9221_7#31 |
| K11 | LT174533 | 5151_6#1 |
| K12 | LT174534 | 10315_6#1 |
| K13 | LT174535 | 9663_7#13 |
| K15 | LT174536 | 9663_8#79 |
| K16 | LT174537 | 10315_6#61 |
| K17 | LT174538 | 9221_7#76 |
| K18 | LT174539 | 5151_6#3 |
| K19 | LT174540 | 10315_6#74 |
| K2 | LT174548 | 9221_7#41 |
| K20 | LT174542 | 9663_8#49 |
| K22 | LT174543 | 5197_7#1 |
| K24 | LT174544 | 10315_6#62 |
| K25 | LT174545 | 9663_7#73 |
| K27 | LT174546 | 9221_7#85 |
| K28 | LT174547 | 10315_6#53 |
| K3 | LT174553 | 9221_7#39 |
| K30 | LT174549 | 5151_3#10 |
| K31 | LT174550 | 9221_7#38 |
| K38 | LT174551 | 5151_3#2 |
| K39 | LT174552 | 5193_5#11 |
| K42 | LT174554 | 5197_2#7 |
| K43 | LT174555 | 5151_2#11 |
| K45 | LT174556 | 9221_7#10 |
| K46 | LT174557 | 9663_7#10 |
| K47 | LT174558 | 5151_2#10 |
| K48 | LT174559 | 10315_6#29 |
| K49 | LT174560 | 5151_6#2 |
| K5 | LT174568 | 5151_2#3 |
| K51 | LT174561 | 9663_7#14 |
| K52 | LT174562 | 9221_7#89 |
| K54 | LT174563 | 5193_7#3 |
| K55 | LT174564 | 5151_3#11 |
| K56 | LT174565 | 5193_3#11 |
| K57 | LT174566 | 9663_8#46 |
| K58 | LT174567 | 9221_7#47 |
| K6 | LT174571 | 9663_8#33 |
| K62 | LT174569 | 5193_2#12 |
| K64 | LT174570 | 9221_7#88 |
| K81 | LT174572 | 9663_8#10 |
| K9 | LT174573 | 5299_7#2 |
| KL103 | LT174574 | 9663_8#47 |
| KL105 | LT174575 | 5193_8#6 |
| KL106-1 | LT174576 | 10315_6#88 |
| KL108 | LT174577 | 5197_2#9 |
| KL109 | LT174578 | 10315_6#32 |
| KL110 | LT174579 | 10315_6#40 |
| KL111 | LT174580 | 10315_6#70 |
| KL112 | LT174581 | 10315_6#79 |
| KL113 | LT174582 | 5151_2#8 |
| KL114 | LT174583 | 5193_5#7 |
| KL115 | LT174584 | 5193_6#2 |
| KL116 | LT174585 | 5197_2#12 |
| KL117 | LT174586 | 5197_8#8 |
| KL118 | LT174587 | 5235_7#7 |
| KL119 | LT174588 | 5299_7#1 |
| KL120 | LT174589 | 5299_7#7 |
| KL121 | LT174590 | 9221_7#19 |
| KL122 | LT174591 | 9221_7#30 |
| KL123 | LT174592 | 9663_8#14 |
| KL124 | LT174593 | 9663_8#56 |
| KL125 | LT174594 | 9663_8#83 |
| KN2 | LT174595 | 10315_6#12 |
