# Supplementary material for: The diversity of Klebsiella pneumoniae surface polysaccharides
Source: Microb Genom. 2016 Aug 25;2(8):e000073. doi: 10.1099/mgen.0.000073 (PMC5320592; doi:10.1099/mgen.0.000073)
Supplement: Supplementary file 4 [file mgen-02-73-s004.docx]

**Supplementary Table S3:**

Sequence accession numbers of virulence genes

| **Gene** | **NCBI Accession: nucleotide position** |
| --- | --- |
| yersiniabactin | CP006738.1: 1835765 to 1866050 |
| aerobactin | FO834905.1: 52175 to 60145 |
| colibactin | CP001969.1: 2192099 to 2246559 |
| salmochelin | CP010316.1: 3692 to 13202 |
| rmpA and rmpA2 [Mucosity regulator] | KF801503.1: 1 to 535  AP006725.1: 3445784 to 3446416  KJ481790.1: 1 to 527  KP760051.1: 1 to 491  (The presence of any of these sequences has been treated as presence of rmpA) |
| fim [type 1 fimbriae] | CP000647.1: 3725589 to 3728183  CP000647.1: 3729003 to 3729776  CP000647.1: 3729790 to 3730368  CP006722.1: 726925 to 727473  CP000647.1: 3730919 to 3731650  CP006722.1: 725704 to 726159  (The presence of the majority of these sequences has been treated as presence of type 1 fimbriae) |
| mrk [type 3 fimbriae] | CP008929.1: 837089 to 837703  CP009876.1: 4289032 to 4289733  CP008929.1: 833794 to 836280  CP008929.1: 832808 to 833803  CP008929.1: 832155 to 832794  (The presence of the majority of these sequences has been treated as presence of type 3 fimbriae) |
