# Supplementary figures and images for: The diversity of Klebsiella pneumoniae surface polysaccharides
Source: Microb Genom. 2016 Aug 25;2(8):e000073. doi: 10.1099/mgen.0.000073 (PMC5320592; doi:10.1099/mgen.0.000073)

A) D-galactan I (O1 and O2)

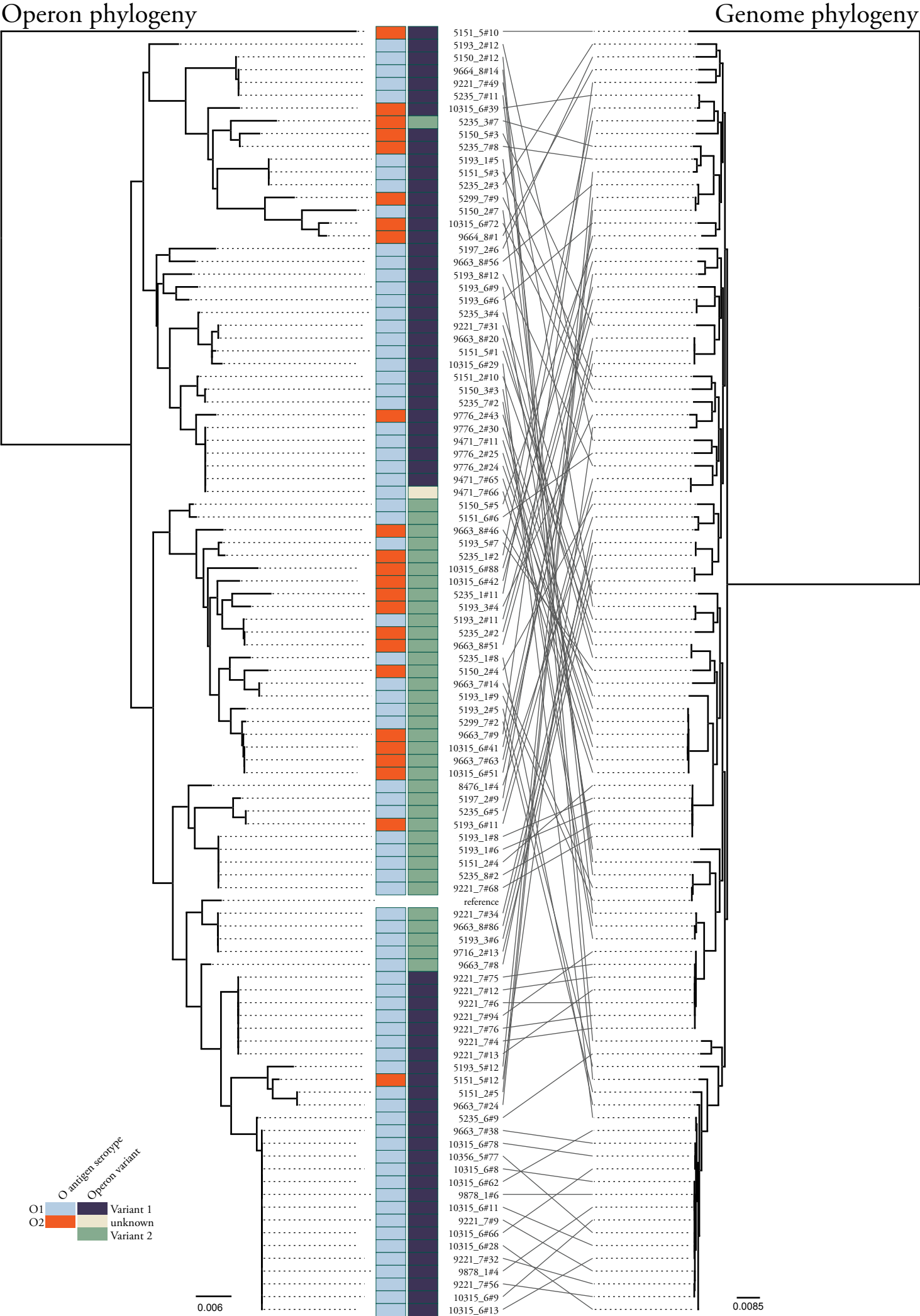

B) O3, O5, and OL104

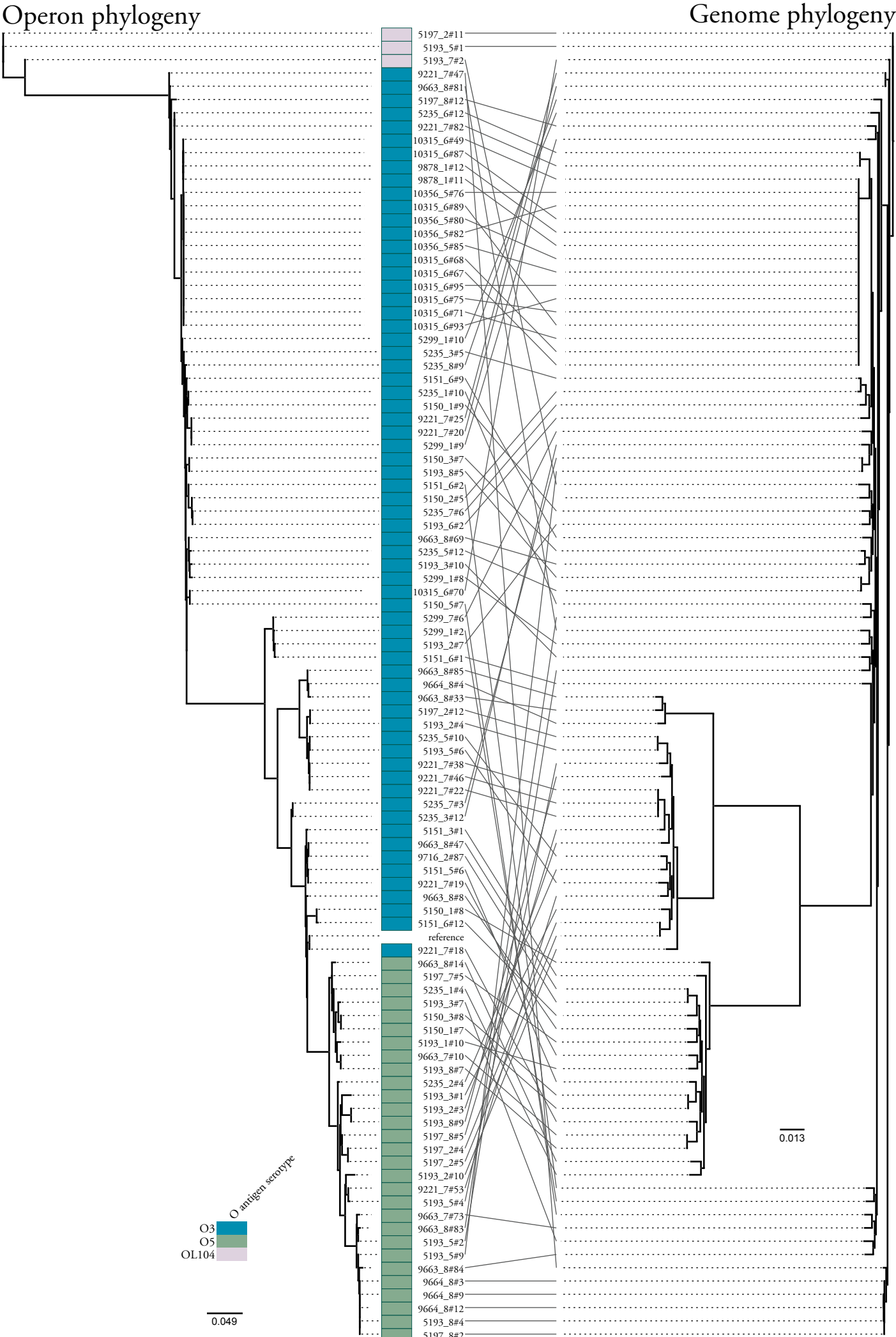

Supplement: Supplementary file 6 [file mgen-02-73-s006.pdf]

A) Phylogeny of *wzm*

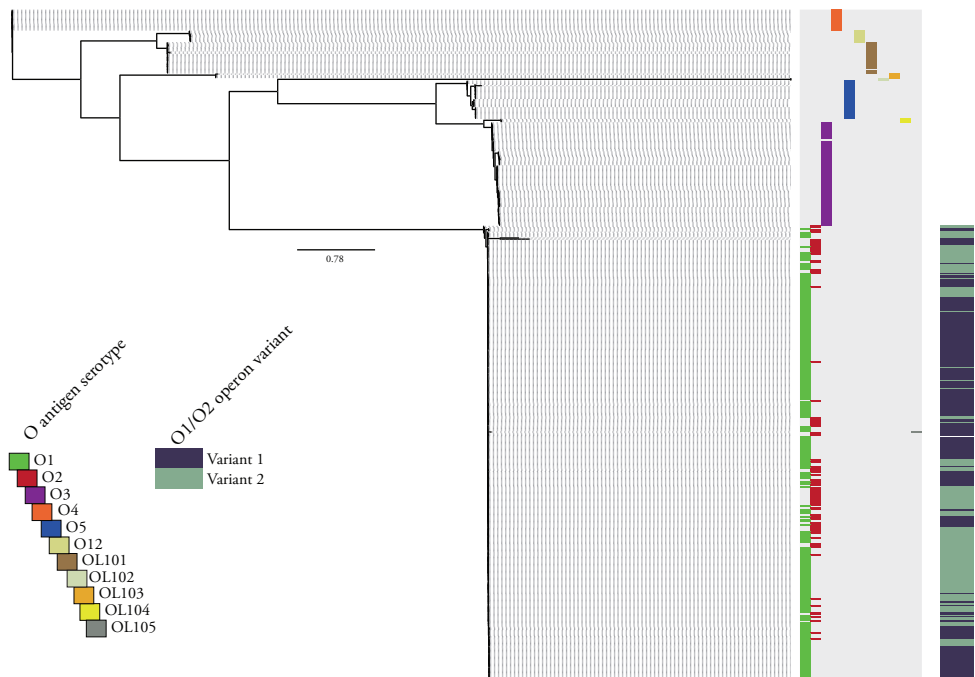

B) Phylogeny of *wzt*

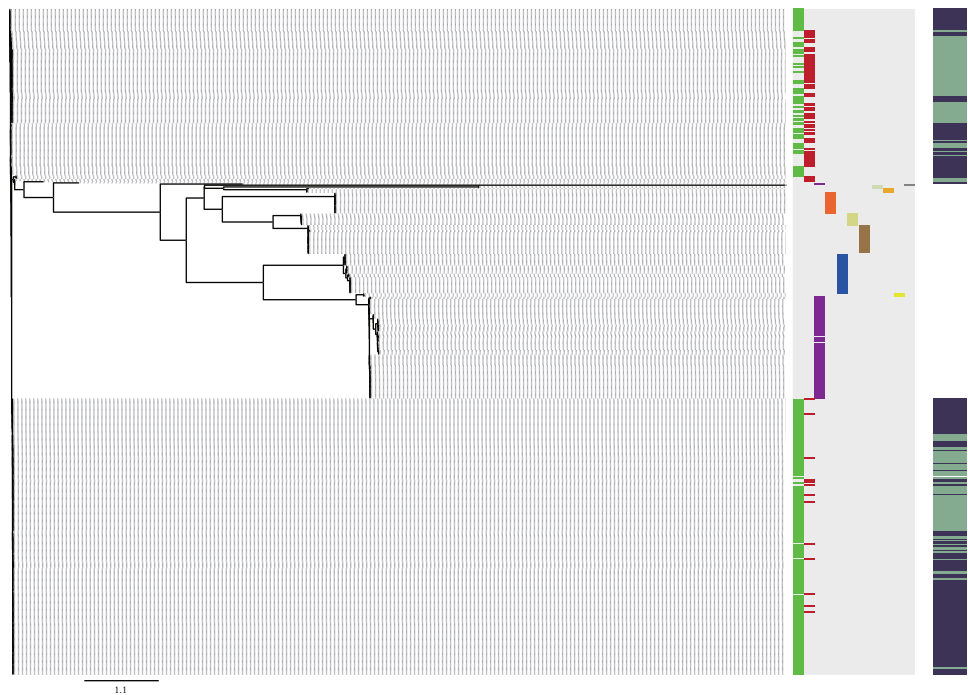

Supplement: Supplementary file 7 [file mgen-02-73-s007.pdf]
