# Supplementary material for: The diversity of Klebsiella pneumoniae surface polysaccharides
Source: Microb Genom. 2016 Aug 25;2(8):e000073. doi: 10.1099/mgen.0.000073 (PMC5320592; doi:10.1099/mgen.0.000073)
Supplement: Supplementary file 5 [file mgen-02-73-s005.pdf]

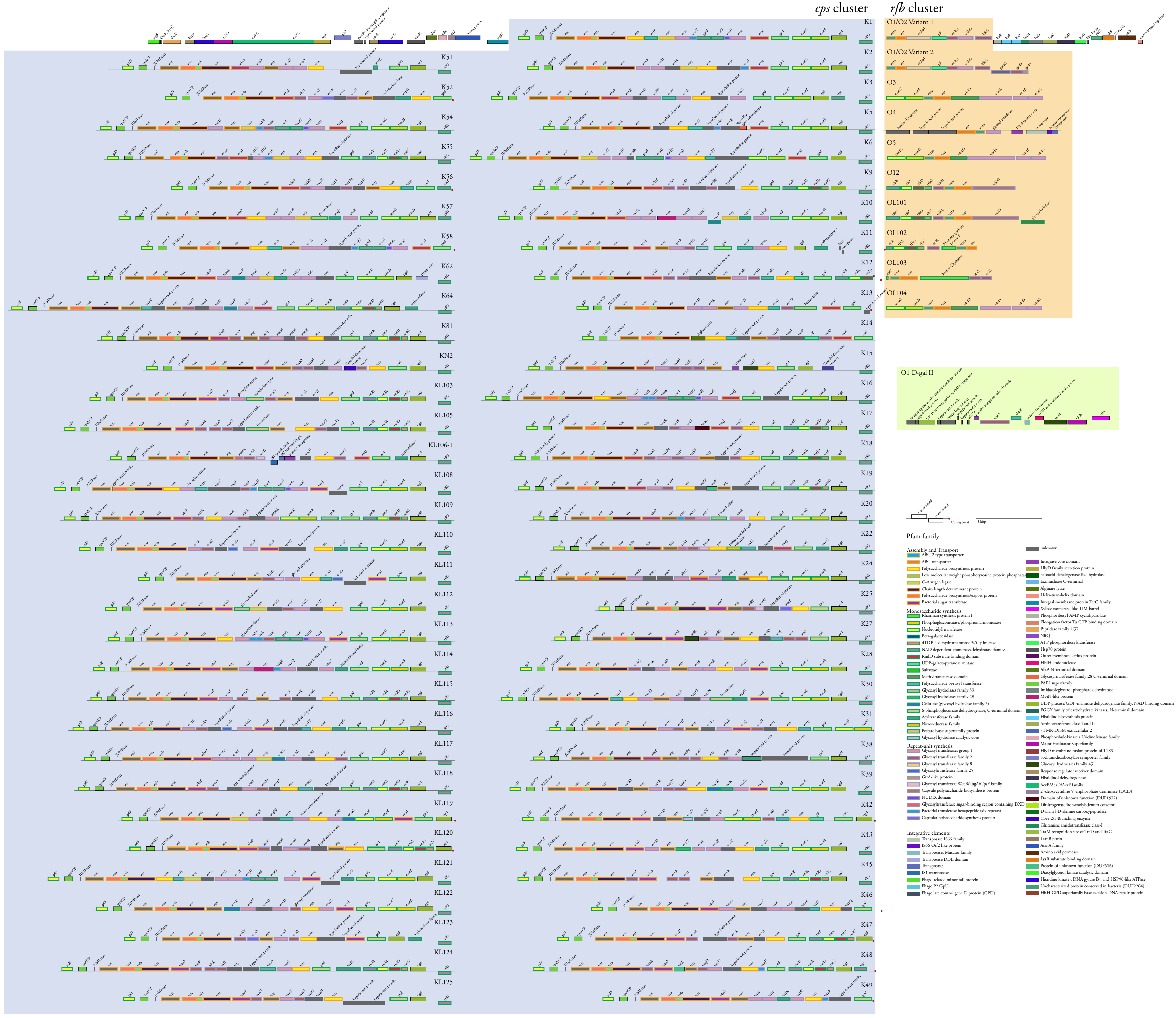

cps cluster
rfb cluster
K1
K2
K3
K5
K6
K9
K10
K11
K12
K13
K14
K15
K16
K17
K18
K19
K20
K22
K24
K25
K27
K28
K30
K31
K38
K39
K42
K43
K45
K46
K47
K48
K49
K51
K52
K54
K55
K56
K57
K58
K62
K64
K81
KN2
KL103
KL105
KL106-1
KL108
KL109
KL110
KL111
KL112
KL113
KL114
KL115
KL116
KL117
KL118
KL119
KL120
KL121
KL122
KL123
KL124
KL125
O1/O2 Variant 1
O1/O2 Variant 2
O3
O4
O5
O12
OL101
OL102
OL103
OL104
O1 D-gal II
Upper strand
Lower strand
Contig break
5 kbp
Pfam family
Assembly and Transport
ABC-2 type transporter
ABC transporter
Polysaccharide biosynthesis protein
Low molecular weight phosphotyrosine protein phosphatase
O-Antigen ligase
Chain length determinant protein
Polysaccharide biosynthesis/export protein
Bacterial sugar transferase
Monosaccharide synthesis
Rhamnan synthesis protein F
Phosphoglucomutase/phosphomannomutase
Nucleotidyl transferase
Beta-galactosidase
dTDP-4-dehydrorhamnose 3,5-epimerase
NAD dependent epimerase/dehydratase family
RmlD substrate binding domain
UDP-galactopyranose mutase
Sulfatase
Methyltransferase domain
Polysaccharide pyruvyl transferase
Glycosyl hydrolases family 39
Glycosyl hydrolases family 28
Cellulase (glycosyl hydrolase family 5)
6-phosphogluconate dehydrogenase, C-terminal domain
Acyltransferase family
Nitroreductase family
Pectate lyase superfamily protein
Glycosyl hydrolase catalytic core
Repeat-unit synthesis
Glycosyl transferases group 1
Glycosyl transferase family 2
Glycosyl transferase family 8
Glycosyltransferase family 25
GtrA-like protein
Glycosyl transferase WecB/TagA/CpsF family
Capsule polysaccharide biosynthesis protein
NUDIX domain
Glycosyltransferase sugar-binding region containing DXD
Bacterial transferase hexapeptide (six repeats)
Capsular polysaccharide synthesis protein
Integrative elements
Transposase IS66 family
IS66 Orf2 like protein
Transposase, Mutator family
Transposase DDE domain
Transposase
IS1 transposase
Phage-related minor tail protein
Phage P2 GpU
Phage late control gene D protein (GPD)
unknown
Integrase core domain
HlyD family secretion protein
haloacid dehalogenase-like hydrolase
Exonuclease C-terminal
Alginate lyase
Helix-turn-helix domain
Integral membrane protein TerC family
Xylose isomerase-like TIM barrel
Phosphoribosyl-AMP cyclohydrolase
Elongation factor Tu GTP binding domain
Peptidase family U32
NirQ
ATP phosphoribosyltransferase
Hsp70 protein
Outer membrane efflux protein
HNH endonuclease
AlkA N-terminal domain
Glycosyltransferase family 28 C-terminal domain
PAP2 superfamily
Imidazoleglycerol-phosphate dehydratase
MviN-like protein
UDP-glucose/GDP-mannose dehydrogenase family, NAD binding domain
FGGY family of carbohydrate kinases, N-terminal domain
Histidine biosynthesis protein
Aminotransferase class I and II
7TMR-DISM extracellular 2
Phosphoribulokinase / Uridine kinase family
Major Facilitator Superfamily
HlyD membrane-fusion protein of T1SS
Sodium:dicarboxylate symporter family
Glycosyl hydrolases family 43
Response regulator receiver domain
Histidinol dehydrogenase
AcrB/AcrD/AcrF family
2'-deoxycytidine 5'-triphosphate deaminase (DCD)
Domain of unknown function (DUF1972)
Dinitrogenase iron-molybdenum cofactor
D-alanyl-D-alanine carboxypeptidase
Core-2/I-Branching enzyme
Glutamine amidotransferase class-I
TraM recognition site of TraD and TraG
LamB porin
AsmA family
Amino acid permease
LysR substrate binding domain
Protein of unknown function (DUF616)
Diacylglycerol kinase catalytic domain
Histidine kinase-, DNA gyrase B-, and HSP90-like ATPase
Uncharacterized protein conserved in bacteria (DUF2264)
HhH-GPD superfamily base excision DNA repair protein
